# Supplementary material for: The interactome of intact mitochondria by cross-linking mass spectrometry provides evidence for coexisting respiratory supercomplexes
Source: Mol Cell Proteomics. 2017 Dec 8;17(2):216–32. doi: 10.1074/mcp.RA117.000470 (PMC5795388; doi:10.1074/mcp.RA117.000470)
Supplement: Supplemental Data [file supp_RA117.000470_133922_0_supp_23978_fzffwf.zip › spectra_annotation/mito_DR_spectra_annotation/33-2-6-1-11-1.pdf]

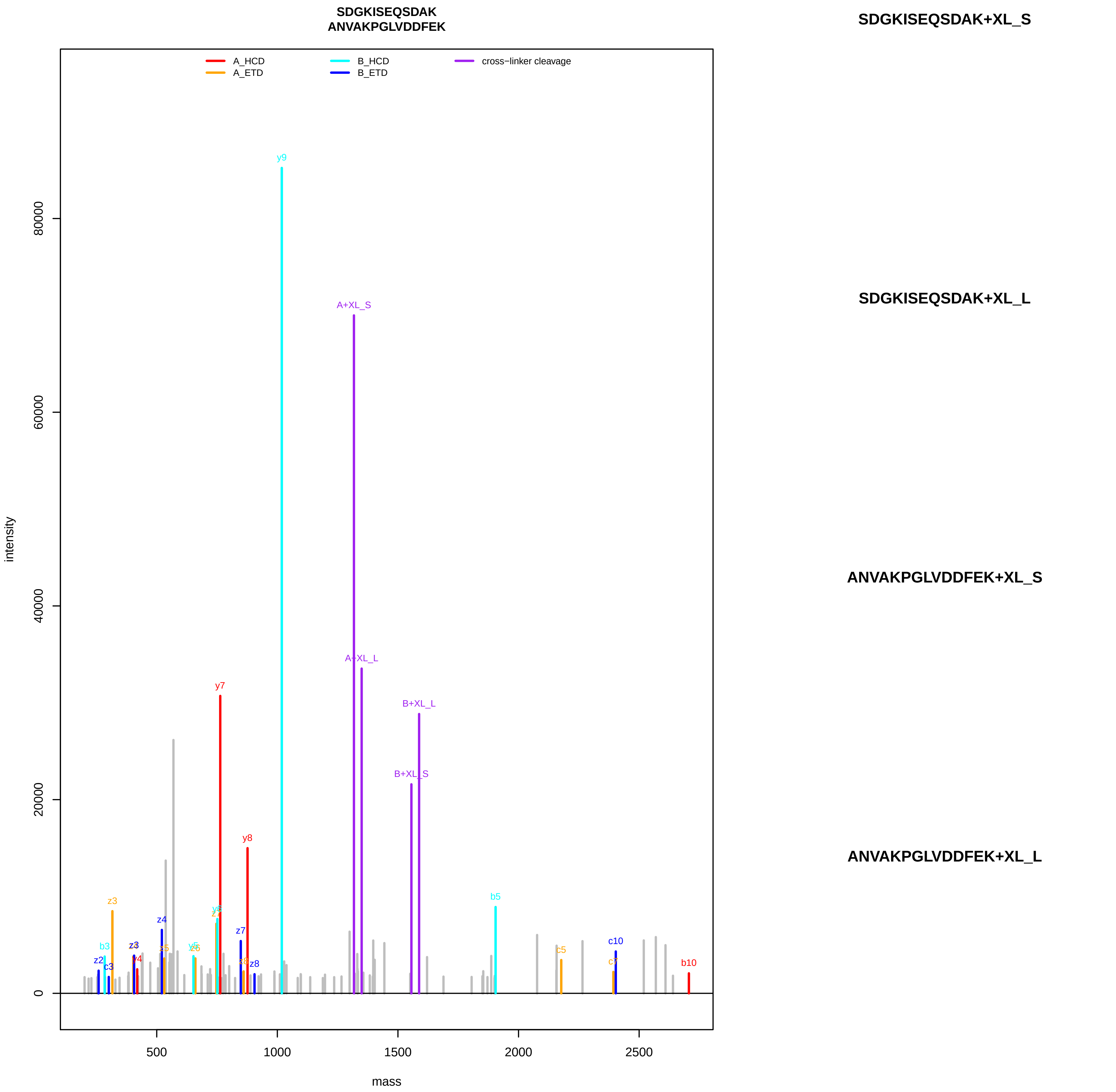
SDGKISEQSDAK
ANVAKPGLVDDFEK
A_HCD
A_ETD
B_HCD
B_ETD
cross−linker cleavage
y9
A+XL_S
A+XL_L
B+XL_L
B+XL_S
y7
y8
b5
z3
z4
z7
z8
c10
c5
c7
b10
z2
b3
c3
y4
y5
z6
z5
intensity
mass
0
20000
40000
60000
80000
500
1000
1500
2000
2500
SDGKISEQSDAK+XL_S
SDGKISEQSDAK+XL_L
ANVAKPGLVDDFEK+XL_S
ANVAKPGLVDDFEK+XL_L
